# D-Propranolol Impairs EGFR Trafficking and Destabilizes Mutant p53 Counteracting AKT Signaling and Tumor Malignancy

**DOI:** 10.3390/cancers13143622

**Published:** 2021-07-20

**Authors:** Jonathan Barra, Javier Cerda-Infante, Lisette Sandoval, Patricia Gajardo-Meneses, Jenny F. Henriquez, Mariana Labarca, Claudia Metz, Jaime Venegas, Claudio Retamal, Claudia Oyanadel, Jorge Cancino, Andrea Soza, Mauricio A. Cuello, Juan Carlos Roa, Viviana P. Montecinos, Alfonso Gonzalez

**Affiliations:** 1 Centro de Biología Celular y Biomedicina (CEBICEM), Facultad de Medicina y Ciencia, Universidad San Sebastián, Santiago 7510157, Chile; barracj@amc.edu (J.B.); lpsandov@uc.cl (L.S.); mlabarcal@docente.uss.cl (M.L.); claudia.metz@uss.cl (C.M.); jvenegasc1@correo.uss.cl (J.V.); claudio.retamal@uss.cl (C.R.); claudia.oyanadel@uss.cl (C.O.); jorge.cancino@uss.cl (J.C.); andrea.soza@uss.cl (A.S.); 2 Centro de Envejecimiento y Regeneración (CARE), Facultad de Ciencias Biológicas, Pontificia Universidad Católica de Chile, Santiago 8330025, Chile; 3 Fundación Ciencia y Vida, Santiago 7780272, Chile; pngajard@uc.cl; 4 Departamento de Hematología-Oncología, Facultad de Medicina, Pontificia Universidad Católica de Chile, Santiago 8330023, Chile; jocerda@uc.cl (J.C.-I.); jfhenriq@uc.cl (J.F.H.); vmontecinos@med.puc.cl (V.P.M.); 5 Departamento de Ginecología-Obstetricia, Facultad de Medicina, Pontificia Universidad Católica de Chile, Santiago 8330023, Chile; macuello@med.puc.cl; 6 Departamento de Patología, Facultad de Medicina, Pontificia Universidad Católica de Chile, Santiago 8330023, Chile; jcroa@med.puc.cl

**Keywords:** EGFR, AKT, p53, HSP90, D-Propranolol, phosphatidic acid, PKA

## Abstract

**Simple Summary:**

Cancer progression is frequently driven by altered functions of EGFR belonging to the tyrosine-kinase family of growth factor receptors and by the transcription factor p53, which is called the “genome guardian”. We report that D-Propranolol, previously used for other purposes in human patients, has antitumor effects involving a redistribution of cell surface EGFR to intracellular compartments and degradation of gain-of-function mutants of p53 (GOF-mutp53). These effects can be seen in cancer cell lines expressing EGFR and GOF-mutp53 and are reproduced in vivo, reducing tumor growth and prolonging survival of xenografted mice. D-Propranolol is proposed as a prototype drug for a new strategy against highly aggressive EGFR- and mutp53-expressing tumors.

**Abstract:**

Cancer therapy may be improved by the simultaneous interference of two or more oncogenic pathways contributing to tumor progression and aggressiveness, such as EGFR and p53. Tumor cells expressing gain-of-function (GOF) mutants of p53 (mutp53) are usually resistant to EGFR inhibitors and display invasive migration and AKT-mediated survival associated with enhanced EGFR recycling. D-Propranolol (D-Prop), the non-beta blocker enantiomer of propranolol, was previously shown to induce EGFR internalization through a PKA inhibitory pathway that blocks the recycling of the receptor. Here, we first show that D-Prop decreases the levels of EGFR at the surface of GOF mutp53 cells, relocating the receptor towards recycling endosomes, both in the absence of ligand and during stimulation with high concentrations of EGF or TGF-α. D-Prop also inactivates AKT signaling and reduces the invasive migration and viability of these mutp53 cells. Unexpectedly, mutp53 protein, which is stabilized by interaction with the chaperone HSP90 and mediates cell oncogenic addiction, becomes destabilized after D-Prop treatment. HSP90 phosphorylation by PKA and its interaction with mutp53 are decreased by D-Prop, releasing mutp53 towards proteasomal degradation. Furthermore, a single daily dose of D-Prop reproduces most of these effects in xenografts of aggressive gallbladder cancerous G-415 cells expressing GOF R282W mutp53, resulting in reduced tumor growth and extended mice survival. D-Prop then emerges as an old drug endowed with a novel therapeutic potential against EGFR- and mutp53-driven tumor traits that are common to a large variety of cancers.

## 1. Introduction

Among proteins whose dysfunctions most frequently contribute to carcinogenesis and tumor progression are the EGFR, which belongs to the ErbB family of tyrosine-kinase receptors that regulate cell proliferation, survival and migration [1,2], as well as the “genome guardian” transcription factor p53 that protects cells against DNA damage and is a tumor suppressor [3,4]. EGFR elevated signaling due to overexpression, mutations, autocrine stimulation or endocytic alterations can contribute to tumor cell malignancy [5,6,7,8,9]. Missense mutations of p53 (mutp53) leading to p53 loss of function promote tumorigenesis and a subset acquires a variety of gain-of-function (GOF) properties, reprogramming cell behavior and promoting cancer progression [3,4,10,11]. Both EGFR and GOF mutp53 impinge “oncogenic addiction” in cancer cells, meaning that these cells can be more selectively damaged through these targets [12]. Indeed, simultaneous counteraction of EGFR and mutp53 oncogenic dysfunctions may improve the therapeutic results against a variety of tumors [13]. Drugs targeting the EGFR activity are in clinical use, but the responses are not optimal and often elicit resistance [1,14,15]. Mutp53 remains an attractive but difficult therapeutic target, still under intense scrutiny [3,4,10,16]. An interesting, yet unexplored opportunity of simultaneously counteracting EGFR and mut53 oncogenic traits is provided by endocytic trafficking, crucial in a variety of cellular processes, including ligand-activated and ligand-independent EGFR functions [5,8,9,17,18]. GOF mutp53-driven cell survival and invasion have been shown to involve an increased EGFR endocytic recycling and signaling [4,19]. Furthermore, a previously described pharmacologic perturbation of EGFR endocytic trafficking results to be deleterious to EGFR-dependent tumor cells [20], and might eventually counteract GOF mutp53-mediated EGFR recycling and signaling [21].

EGFR has been a paradigm of endocytosis induced by ligand and signaling regulated by vesicular trafficking along recycling or degradation routes [5,6,7,8,9]. Upon ligand binding, the EGFR dimerizes, its intracellular tyrosine-kinase becomes activated and phosphorylates the receptor at several tyrosines, which constitute signaling initiators [22] and promote the endocytosis of active EGFR [5]. EGFR signaling includes ERK and AKT pathways, which are often found increased in tumor cells [21,23]. The intracellular trafficking of EGFR depends on the concentration and kind of stimulating ligand [24,25], as well as concomitant conditions such as the overexpression of certain endocytic proteins [23] or GOF mutp53 [19,21]. High EGF concentrations promote EGFR endocytic sorting towards lysosomal degradation that attenuates signaling [5,7]. However, within tumors, low EGF concentrations [26], stimulation with TGF-α even at high concentrations [25], or expression of GOF mutp53 [19], can drive activated EGFR instead to endocytic recycling routes [5,7], which prolong and enhance signaling, particularly via ERK and AKT pathways that promote cell proliferation, survival and invasive migration [19,27,28,29].

*TP53* is the most frequently mutated gene in human cancers [3,4,16]. Its encoded transcription factor p53 is normally maintained at low levels due to proteasomal degradation after ubiquitination by E3 ubiquitin ligases such as MDM2 and CHIP [30]. Stress conditions, including DNA damage, stabilize p53 by promoting a protective interaction with the chaperone machinery that includes HSP90/HDAC6 proteins [30,31]. Activated p53 arrests the cell cycle and increases the expression of genes involved in DNA repair, or otherwise stimulates apoptosis or senescence to exclude cells with irreparable genetic damage [16,32]. Loss of *TP53* increases the possibility of accumulating mutations that enhance tumor progression [33]. 

However, missense mutations mainly located within the DNA binding domain (DBD) of p53 are the most frequent and include hotspots for mutations, such as R175, R273, R245, R248 and R282W, which stabilize p53 with a loss of wild-type tumor suppression function, acquiring GOF properties and high levels of expression [3]. Cancer cells become addicted to the continued presence of high levels of GOF mutp53, thus offering therapeutic opportunities with minimal damage of normal cells [10]. GOF mutp53 modify a variety of molecular pathways that contribute to cell survival, proliferation, invasive migration and resistance to current therapies, among other effects [10]. Therefore, predominant therapeutic strategies under intense scrutiny include attempts to promote destabilization and subsequent degradation of GOF mutp53 proteins, e.g., inhibiting the HSP90 axis [10,34]. An interesting alternative might be to target the enhanced endocytic recycling of growth factor receptors promoted by GOF mutp53 [4,21]. GOF mutp53 stimulates EGFR recycling coupled to α5β1 integrin, which increases AKT signaling, tumor cell survival, invasion and metastasis [19,35]. In principle, the pharmacologic arrest of EGFR at endosomes might counteract cancer malignancy driven by EGFR and GOF mutp53 proteins.

Phosphatidic acid (PA) provides a druggable signaling pathway that can be used to promote EGFR endocytosis and arrest at recycling endosomes [36], with selective deleterious effects against several EGFR-dependent cancer cells [20]. The inhibition of PA phosphohydrolase (PAP) activity increases the levels of PA leading to the activation of type-4 phosphodiesterases (PDE4), the main regulators of cAMP levels [37]. The consequential decrease of cAMP levels and protein kinase A (PKA) activity leads to EGFR internalization and the accumulation at perinuclear recycling endosomes [36]. These mimic the effects of PKA inhibitors that long ago involved PKA in an unsuspected mechanism of EGFR endocytosis [38]. Interestingly, the PA/PDE4/PKA pathway can be triggered by D-Propranolol (D-Prop) [20], which lacks the beta-blocking activity of its isomer L-propranolol [39,40], and has been experimentally used for other purposes in human patients [41].

Here we found that D-Prop induces both the internalization and accumulation/arrest of EGFR at recycling endosomes, significantly reducing AKT activation, cell proliferation and invasive migration in GOF mutp53 cells, despite their resistance to EGFR tyrosine-kinase inhibitors (TKIs). Strikingly, we unexpectedly found that D-Prop induces proteasome-dependent degradation of different GOF mutp53 proteins due to destabilization of mutp53-HSP90 complex, seemingly related with HSP90 as PKA substrate [42]. We reproduced most of these effects in mutp53 tumor xenografts. A single daily dose of D-Prop significantly reduced tumor growth and improved the survival of NSG mice xenografted with G-415 p53 R282W gallbladder cancer (GBC) cells, accompanied with an increased EGFR internalization-arrest at recycling endosomes and reduced mutp53 protein levels. Therefore, therapies against highly aggressive mutp53-expressing tumors may be improved by D-Prop, even when used at lower doses than previously reported in human patients [41].

## 2. Materials and Methods

Antibodies and reagents information are provided in Appendix A.

### 2.1. Cell Culture

Cancer cell lines: G-415 (gallbladder cancer) (Riken BioResource Center, Ibaraki, Japan), EIH1299 (non-small cell lung cancer) expressing p53 R273H in a ponasterone A inducible manner (kindly provided by Karen Vousden, Francis Crick Institute, London, UK) [43] and Panc-1 (pancreatic ductal adenocarcinoma) (ATCC, Manassas, VA, USA) were grown on DMEM F-12 supplemented with 10% heat-inactivated fetal bovine serum (HyClone, Logan, UT, USA), 100 units/mL penicillin and 100 μg/mL streptomycin at 37 °C in a humidified atmosphere containing 5%-CO_2_.

### 2.2. Indirect Immunofluorescence

Immunofluorescence experiments were performed as previously described [36], acquiring images with a Leica TCS SP8 spectral confocal microscopy (63X oil immersion objective), processing with ImageJ software (NIH) and colocalizing analysis were performed using JaCoP plugin [44].

### 2.3. EGFR Endocytosis

Surface and internalized EGFR was assessed by flow cytometry as previously described [36]. Details are given in the Appendix A.

### 2.4. Cell Viability and Apoptosis

Cell viability was determined by the MTT (3-(4,5-dimethylthiazol-2-yl)-2,5-diphenyl tetrazolium bromide) assay (Sigma-Aldrich, St. Louis, MO, USA) according to protocols previously described [45]. Apoptosis and cell death were assessed using the Annexin V Apoptosis Detection Kit FITC (Thermo Fisher, #88-8005, Waltham, MA, USA) according to manufacturer instructions, and FACScalibur Flow Cytometer with CellQuest software (BD Biosciences, San Jose, CA, USA).

### 2.5. Inverted Invasion Assay

Inverted invasion assay through Matrigel (Corning, #35423, Corning, NY, USA) enriched in fibronectin and using EGF as chemoattractant was performed in 24-well Transwell™ polycarbonate filters 8-μm pore size inserts (Corning, #3422, Corning, NY, USA) as previously described [46,47]. Details are described in the Appendix A.

### 2.6. Immunoprecipitation and Immunoblotting

Standard procedures were used as previously described [48]. See details in Appendix A.

### 2.7. PA Levels and PKA Activity Biosensors

G-415-R282W cells grown on 350-mm MatTek glass bottom dishes (MatTek Corp.) were transiently transfected with TransIT-X2 (Mirus Bio, Madison, WI, USA) to express the biosensors RFP-PASS for PA levels [49] (kindly provided by Guangwei Du, University of Texas) and ExRAi-AKAR for PKA activity [50] (kindly provided by Dr. Jin Zhang, UCSD). After 24 h, the cells were incubated on live-cell record media (phenol red-free DMEM, 10 mM Hepes, pH7.4) and images were acquired every 10 s for 20 min on a SP8 Leica Spectral Confocal Microscope (63X 1.4 N.A. oil immersion objective). PA level changes are reflected by the fluorescence intensity (F) under 561 nm laser excitation normalized to t0 ((Ftx/Ft0)/Ft0). PKA activity was assessed through FRET changes quantitated by the CFP/YFP ratio (R) normalized to t0 ((Rtx/Rt0)/Rt0). Image analysis and quantification was performed using ImageJ software (NIH).

### 2.8. TP53 Targeted Sequencing

Genomic DNA was extracted from G-415 cells using DNAzol (Thermo Fisher Scientific) according to manufacturer protocol and specific DNA sequences were amplified for exons 2-11 of human *TP53*, according to protocols of IARC TP53 Database (https://p53.iarc.fr, accessed on 31 August 2018). PCR products were cloned on pGEM^®^-T Easy Vector (Promega, Madison, WI, USA) for sequencing (Macrogen Corp., Seoul, Korea), according to manufacturer instructions.

### 2.9. Subcutaneous Xenografts

Mice experiments of G-415 R282W xenografts were performed in accordance with and approved by the Institutional Animal Care and Use Committee (IACUC) at the Faculty of Medicine of Pontificia Universidad Católica de Chile. Details on xenografts generation, D-Prop treatment, EGFR internalization and immunohistochemical analysis of tumors are described in the Appendix A.

### 2.10. Statistical Methods

Unpaired two-tailed Student *t*-tests (for two datasets) or one-way ANOVA with Tukey’s post-hoc test (for multiple datasets) were used for data analyses. Data presented are means ± SEM. All statistical tests were justified as appropriate. All experiments were performed as a minimum of three independent replicates. Survival was calculated using the Kaplan–Meier method (log-rank test). *p*-values less than 0.05 were considered statistically significant (* *p* < 0.05); (** *p* < 0.01). Statistical values were calculated using GraphPad Prism 6.0 (GraphPad Software Inc., San Diego, CA, USA).

## 3. Results

### 3.1. D-Prop Induces Internalization of EGFR in Tumoral Cells Expressing GOF mutp53

PAP inhibition with drugs such as propranolol or its L and D isomers has been shown to induce EGFR internalization in several cancer cell lines involving increased endocytosis and recycling arrest [20,36]. Therefore, we first assessed whether a similar EGFR internalization and accumulation at the recycling endosomes is reproduced in cells expressing GOF mutp53, as in these cells an increased recycling of EGFR can occur associated with α5β1-integrins, promoting invasive migration [19]. 

**Figure 1:**
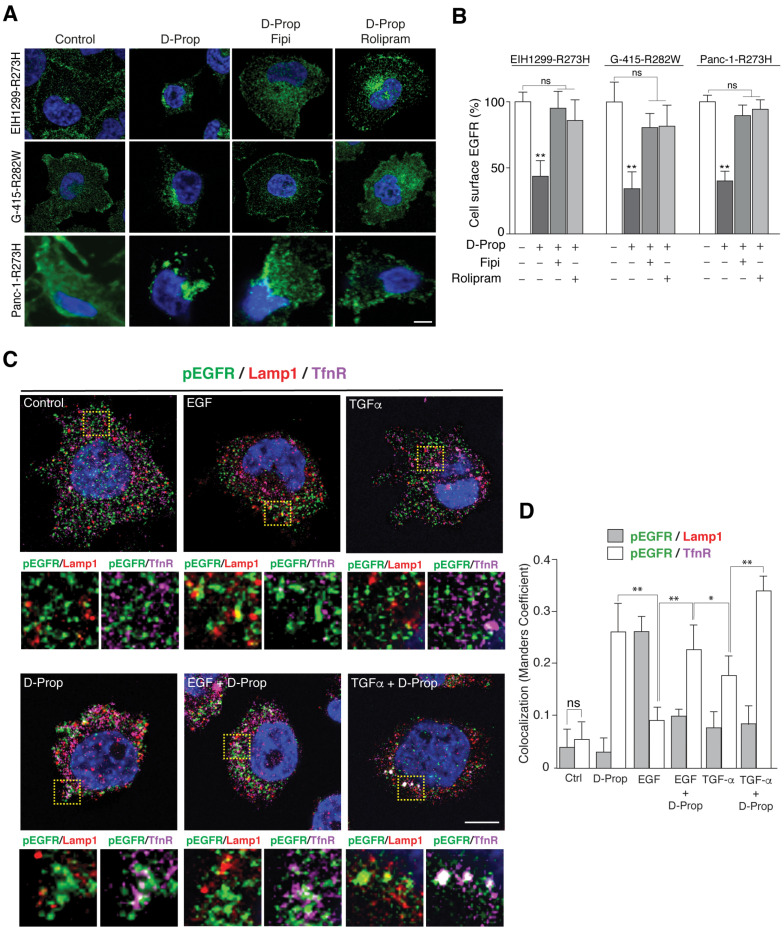
D-Prop induces ligand-independent EGFR internalization and diverts pEGFR stimulated by EGF and TGF-α towards perinuclear recycling endosomes. (**A**,**B**) EGFR redistribution by the PA/PDE4/PKA pathway triggered with D-Prop. (**A**) Indirect immunofluorescence of EGFR (green) in EIH1299-R273H, G-415-R282W and Panc-1-R273H tumor cells treated with D-Prop in the absence or presence of FIPI or Rolipram for 30 min. (**B**) The graph shows the percentage of EGFR at the cell surface assessed by flow cytometry. (**C**,**D**) EGFR colocalization with recycling endosomes and lysosomes markers. (**C**) G-415-R282W tumor cells were treated for 30 min with either EGF or TGF-α (100 ng/mL) in the presence or absence of D-Prop (100 μM), as indicated. Panel shows the triple-immunofluorescence of tyrosine phosphorylated EGFR (pEGFR; green), Lamp1 (red) and TfnR (magenta) and the graph (**D**) illustrates the corresponding colocalization Mander’s coefficient (*n* = 15 cells per condition; Bars graphs shows means ± SEM; * *p* < 0.05; ** *p* < 0.01; one-way ANOVA with Tukey’s post hoc test). Scale bar = 10 μm. ns: not significant.

We chose the following cell lines: non-small cell lung cancer (NSCLC) EIH1299-R273H cell line that is inducible for p53 R273H expression, gallbladder cancer cells G-415, in which we found a p53 R282W mutant (G-415-R282W) and pancreatic ductal adenocarcinoma (PDAC) Panc-1 cells that endogenously express p53 R273H (Panc-1-R273H), all belonging to aggressive tumors [19,51,52]. R273H and R282W correspond to “DNA-contact” and “conformational” hotspot mutations, respectively, within the DNA binding domain [53,54]. Both kind of mutations alter the binding of the p53 protein to its regulated gene regions in the DNA but through different mechanisms. One mutant affects directly the residues involved in the DNA binding while the other determine conformational alterations in the DNA-binding pocket, respectively [55]. D-Prop used at the IC50 for EGFR internalization in HeLa cells [20,36], redistributed EGFR to perinuclear endosomes within 30 min in these cells (Figure 1A). As a result of this internalization, cell surface EGFR decreased 44% in EIH1299-R273H, 35% in G-415-R282W and 51% in Panc-1-R273H cells (Figure 1B). This effect can be counteracted by the inhibition of PLD-mediated PA generation with FIPI and PDE4-mediated degradation of cAMP with Rolipram (Figure 1B), hallmarks of the PA/PDE4/PKA pathway [20,36]. Therefore, these results reproduce the effects of PAP inhibition previously reported in other cancer cells [20,36]. 

**Figure 2:**
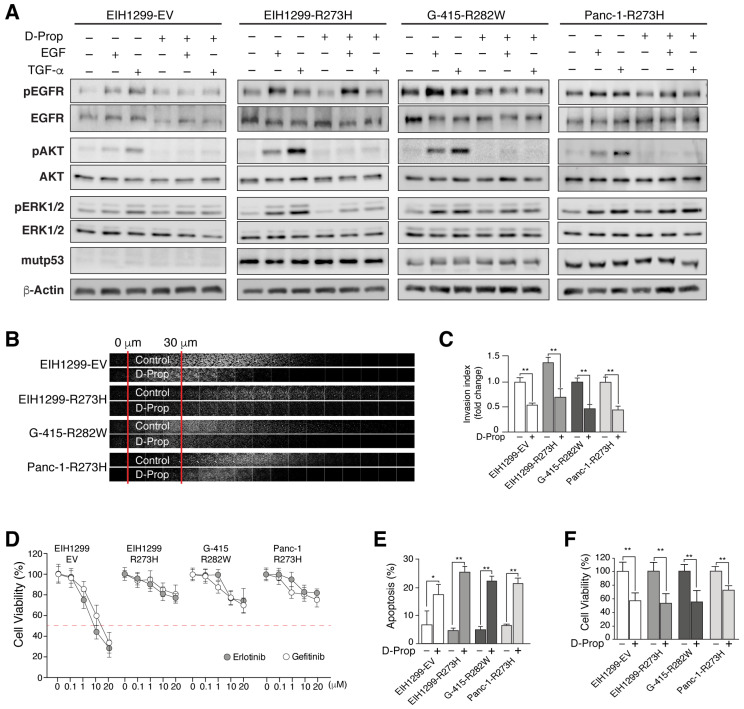
D-Prop impairs EGFR downstream signaling and decreases invasion and viability in GOF mutp53-expressing cells resistant to EGFR tyrosine-kinase inhibitors (TKIs). (**A**) Immunoblot analysis of EGFR, AKT and ERK and their phosphorylated active forms in cells treated with EGF or TGF-α in the presence or absence of D-Prop for 30 min. D-Prop remarkably abrogated AKT responses in all tested mutp53 cells. mutp53-R273H expression induced in EIH1299 cells increased the AKT and ERK activation by EGF and even more by TGF-α, which was also inhibited by D-Prop. (**B**) EIH1299 cells not expressing mutp53 (EV: empty vector) or induced with PonA to express mutp53 R273H, as well as G-415-R282W and Panc-1-R273H tumor cells were subjected to the inverted invasion assay in polycarbonate filters coated with Matrigel plus fibronectin (2.5 g/mL) and incubated with or without D-Prop (100 μM), using 20 ng/mL EGF as chemoattractant for 72 h. Filters were fixed, permeabilized and stained using Hoechst staining. Confocal sections after staining are shown depicting the invasion extension. (**C**) Graph shows the invasion index as fold changes over 30 μm marked between red lines. (**D**) Resistance to EGFR TKIs. Viability was assessed by MTT assay after Erlotinib and Gefitinib treatments at logarithmic increasing concentrations for 72 h. Red dashed line marks resistance threshold considered at IC50 > 20 μM. Only EIH1299 EV cells that do not express mutp53 show sensitivity to both EGFR inhibitors. (**E**,**F**) Cell viability and apoptosis under intermittent D-Prop treatment. Cells were treated with D-Prop (100 μM) for 1 h twice a day with an interval of 6 h during the day for 72 h. Cell viability and apoptosis was assessed by FACS using Annexin V-FITC kit (BD Biosciences). Bars graphs shows means ± SEM. * *p* < 0.05; ** *p* < 0.01; *t*-test.

We then tested the effect of D-Prop on GOF mutp53-expressing cells stimulated with high concentrations of EGF or TGF-α, conditions that target EGFR towards lysosomes or recycling routes, respectively [24,25]. We used G-415-R282W cells that express higher levels of EGFR (See Figure 2A). Double indirect immunofluorescence with an antibody to phosphorylated Tyr-1068 shows that EGF-activated pEGFR co-distributed with Lamp1 rather than TfnR, indicating sorting to late endosomes and lysosomes (Figure 1C,D). In contrast, TGF-α increased pEGFR colocalization with TfnR rather than with Lamp1, reflecting main trafficking through recycling endosomes, as described in other cells [25]. D-Prop increased the colocalization of EGFR with TfnR mostly in perinuclear endosomes (Figure 1C,D). Transferrin and TfnR are widely used markers to detect cargo proteins in the recycling endosomal compartment [56,57,58,59]. Under this condition of D-Prop treatment, we also detected approximately 25–30% of the EGFR colocalizing with Rab11 (Figure S1), another widely used marker of recycling endosome compartment [60,61,62]. Therefore, D-Prop promotes internalization and accumulation in recycling endosomes of the EGFR activated by ligand also in GOF mutp53 cells.

### 3.2. D-Prop Decreases AKT and ERK Signaling, Invasive Migration and Viability of GOF mutp53-Expressing Cells

One of the consequences of EGFR stimulated recycling seen in GOF mutp53 cells is an enhanced EGFR activation and signaling mostly through the AKT pathway [19,35]. Accordingly, EIH1299-R273H cells compared with EIH1299-EV cells that do not express mutp53 responded to both EGF and TGF-α displaying higher activation of EGFR, ERK and AKT (Figure 2A). D-Prop did not affect the activation of the EGFR in any of the mutp53 cells, but nevertheless decreased the activation of both ERK and AKT. The decrease in ERK activation depended on the cell line, mostly affecting EIH1299-R273H and G-415-R282W but not Panc-1-R273H cells (Figure 2A and Figure S2). More remarkable was an almost complete abrogation of AKT activation in all mutp53 cell lines (Figure 2A). Therefore, D-Prop effectively counteracts signaling pathways associated with invasive migration, survival and malignancy of mutp53-expressing cells [19,35].

As might be expected for EGFR internalization and accumulation in recycling endosomes, D-Prop reduced the invasive migration of EIH1299-R273H, G-415-R282W and Panc-1-R273H tumor cells that were tested in fibronectin-enriched Matrigel using complete cell culture media supplemented with EGF as chemo-attractant (Figure 2B,C). Then we tested whether D-Prop might also overcome the resistance to first-line EGFR tyrosine kinase inhibitors (TKIs), Gefitinib and Erlotinib, described as another property conferred by p53 missense mutations [63]. We observed resistance to TKIs developed after mutp53 induction in EIH1299-R273H cells, as well as an intrinsic property of G-415-R282W and Panc-1-R273H tumor cells (Figure 2D). In these cells, D-Prop treatment twice a day for 1 h decreased cell viability and increased apoptosis (Figure 2E,F). We chose this intermittent protocol because previous studies showed that prolonged incubation has similar results on proliferation but the EGFR is no longer found internalized, leaving uncertain the effective period of drug effect [20]. Moreover, the intermittent treatment relates better with the rapid metabolization and short half-life of D-Prop in vivo [41]. Thus, these results show that D-Prop induces EGFR internalization and counteracts invasive migration and resistance to EGFR TKIs driven by GOF mutp53.

### 3.3. D-Prop Induces Mutp53 Proteasomal Degradation through PKA Dependent Destabilization of Mutp53-HSP90 Complex

**Figure 3:**
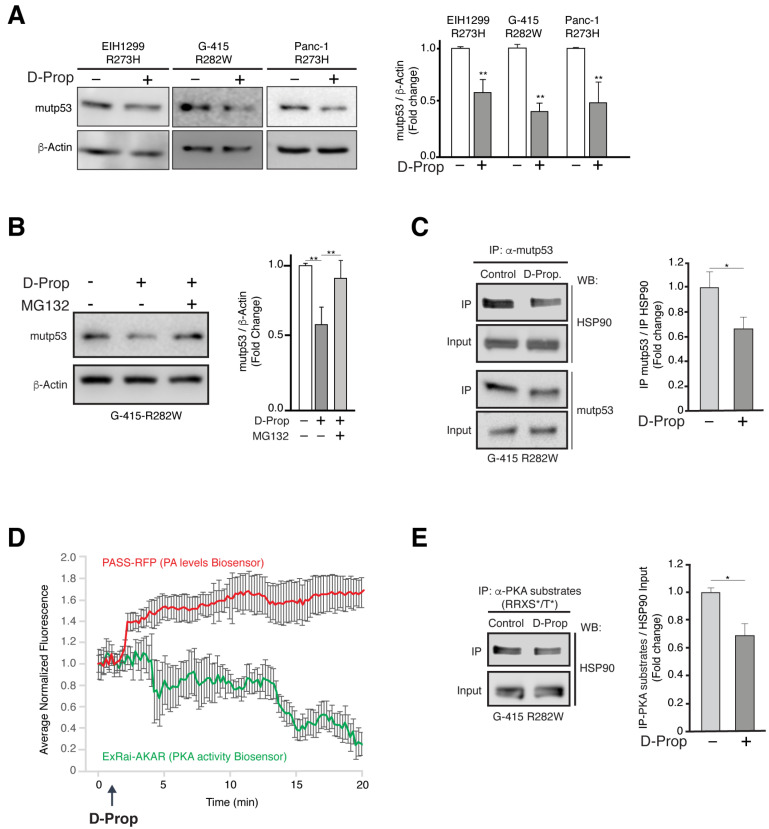
D-Prop decreases mutp53 protein levels involving a destabilization of HSP90-mutp53 complex. (**A**) Effect of D-Prop on mutp53 levels. Cells treated for 1 h with D-Prop (100 μM) showed reduced levels of mutp53 analyzed by immunoblot 24 h later. (**B**) Proteasome-mediated mutp53 degradation induced by D-Prop. G-415-R282W cells treated as in (**A**) and incubated with MG132 20 μM for 1 h show recovered mutp53 levels. (**C**) mutp53 association with HSP90. G-415-R282W cells were incubated with D-Prop (100 μM) for 1 h and then subjected to immunoprecipitation of mutp53 followed by immunoblot of HSP90. D-Prop treatment decreases the co-immunoprecipitation of HSP90 indicating its lower association with mutp53. (**D**) Biosensors show the increase in PA levels followed by the decrease of PKA activity elicited by D-Prop (100 µM) treatment in G-415 R282W cells transiently transfected with the corresponding PASS-RFP and ExRAi-AKAR plasmids. (**E**) Immunoprecipitation using an antibody that recognizes PKA substrates shows that PKA dependent phosphorylation of HSP90 is reduced by D-Prop. Bars graphs shows means ± SEM * *p* < 0.05; ** *p* < 0.01; *t*-test.

Mutp53 levels are characteristically stabilized through an interaction with HSP90 that protects mutp53 from proteasomal degradation [10]. Unexpectedly, we found that D-Prop (100 μM) treatment for 1 h decreased close to 50% the mutp53 protein levels assessed 24 h later (Figure 3A and Figure S3). This effect included the EIH1299-R273H cells that express mutp53 under an exogenous promoter stimulated by ponasterone A, thus making a transcriptional effect unlikely. Instead, the proteasome inhibitor MG132 recovered the protein levels of mutp53 (Figure 3B and Figure S3), indicating that D-Prop induces proteasome-dependent mutp53 degradation and suggesting an interference with the protective role of HSP90. Accordingly, co-immunoprecipitation experiments showed that D-Prop decreased the interaction of mutp53 with HSP90 (Figure 3C and Figure S3). These results indicate that D-Prop induces proteasomal dependent mutp53 degradation and also decreases mutp53-HSP90 interaction.

HSP90 is a PKA substrate [42] and D-Prop indirectly decreases basal PKA activity by triggering the PA/PDE4/PKA pathway [36]. Therefore, we explored the possibility that D-prop treatment leading to PKA inhibition decreases the PKA phosphorylation of HSP90. Biosensors for PA levels [49] and PKA activity [50] transfected in G-415-R282W cells showed the reciprocal changes expected for the activation of the PA/PDE4/PKA pathway, i.e., increments of PA levels followed by decreased PKA activity (Figure 3D). We next tested if HSP90, as recognized substrate of PKA [42], becomes less phosphorylated at the PKA target motifs R-R-X-S/T. Immunoprecipitation with the Phospho-PKA Substrate (RRXS*/T*) antibody followed by immunoblot of HSP90 showed a decreased PKA-mediated HSP90 phosphorylation (Figure 3E and Figure 3S). Therefore, the PA/PDE4/PKA pathway triggered by D-Prop induces proteasome-dependent mutp53 degradation by destabilizing the protective interaction of HSP90 with mutp53, which seems to be regulated by PKA-dependent HSP90 phosphorylation.

### 3.4. D-Prop Decreases Tumor Growth and Extends Free-Survival of G-415-R282W Xenograft-Bearing Mice

**Figure 4:**
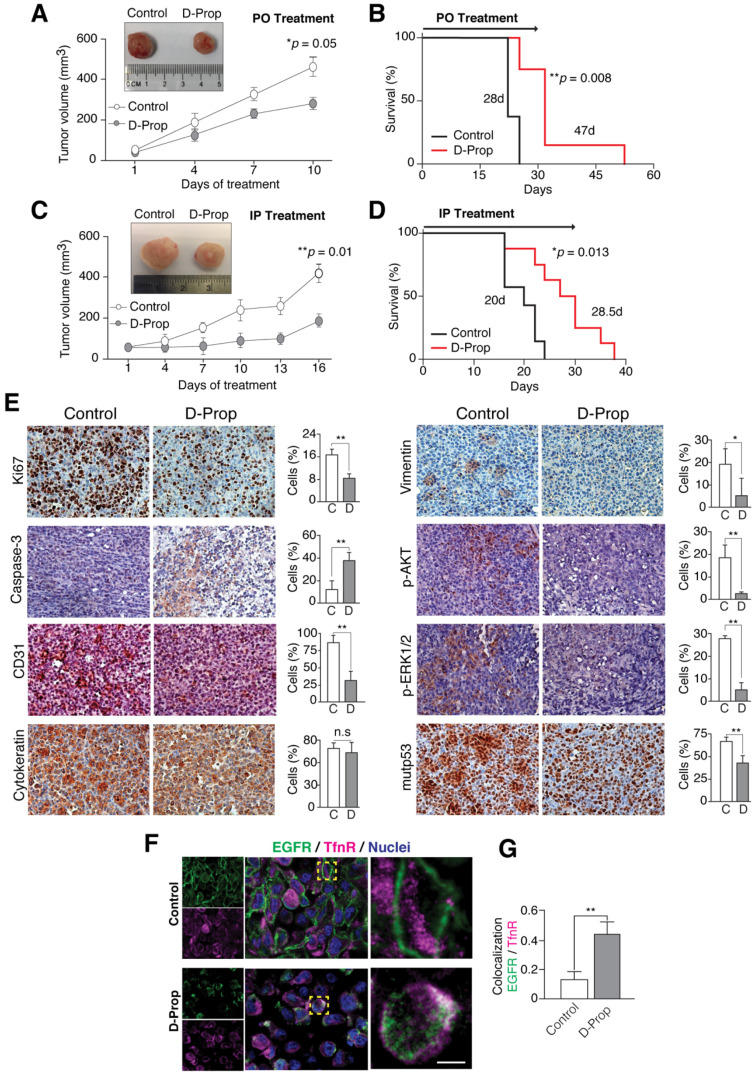
D-Prop inhibits tumor growth and extends the survival of G-415 R282W xenografted NSG mice, with congruent immunohistochemical changes. Treatment started when G-415 R282W xenografts reached 50 mm^3^ and consisted on single daily doses of D-Prop administered for 15 days, either per oral (PO) (70 mg/kg/day) (**A**,**B**) or intraperitoneal (IP) (20 mg/kg/day) (**C**,**D**), as indicated. (**A**,**C**) Tumor volume curves reflect decreased tumor growth under both D-Prop treatments, either PO (* *p* = 0.05, *n* = 5 mice per group) or IP (** *p* = 0.01; *n* = 10 mice per group). Insets show representative images of tumors. (**B**,**D**) Kaplan-Meier analysis shows extended survival under both D-Prop treatments, PO (**B**) (median survival = 47 versus 28 days; *p* = 0.008 log-rank test; *n* = 5) and IP (**D**) (median survivals = 28.5 versus 20 days; *p* = 0.013, log-rank test; *n* = 10). (**E**) Immunohistochemistry (IHC) of tumors at the end of IP D-Prop treatment. IHC images (left and right panels) and graphs with the percentage of cells stained for the indicated proteins show decreased levels of the proliferation marker Ki67, endothelial marker CD31, mesenchymal marker vimentin, signaling proteins pAKT and pERK and mutp53, whereas the apoptotic marker cleavage caspase-3 increased. Cytokeratin show no changes. Graphs shows means ± SEM (* *p* < 0.05; ** *p* < 0.01; *t*-test). (**F**) D-Prop induces EGFR internalization in G-415 R282W xenografts. Immunofluorescence of EGFR (green) with TfnR (magenta) in cryostat sections of G-415 R282W xenografts in NSG mice after 1 h of D-Prop IP administration (20mg/kg/day). Cells in the control xenografts show most EGFR distributed at cellular borders whereas in mice that received a single dose of D-Prop the cells show instead perinuclear EGFR location- (**G**) EGFR colocalization with TfnR by Manders coefficient analysis (25 cells per condition) reflects D-Prop-induced distribution towards recycling endosomes. Bars graphs shows mean ± SEM. ** *p* < 0.01; *t*-test. Scale bar = 10 μm. n.s: not significant.

Finally, we tested D-Prop effectiveness against tumor growth in vivo. As gallbladder cancer (GBC) is highly malignant and resistant to chemotherapy we used the G-415-R282W cells to generate rapidly developed subcutaneous tumors in NOD scid gamma (NSG) mice [52]. Even when propranolol racemic mixture has a short half-life of about 4h in serum [64,65], a single daily dose of D-Prop, either per oral (PO) (70 mg/Kg/day) or intraperitoneal injection (IP) (20 mg/kg/day), not only inhibited tumor growth (Figure 4A,C) but also extended free-survival of xenografted NSG mice (Figure 4B,D). Immunohistochemistry of the tumors examined after 15 days of daily IP D-Prop treatment showed lower levels of Ki67 (proliferation index marker), CD31 (angiogenesis index marker), pAKT and pERK (EGFR signaling markers), vimentin (epithelial-mesenchymal-transition marker), and remarkably, mutp53 levels became ostensibly decreased. Furthermore, cleaved caspase-3 indicated an increased apoptosis (Figure 4E). Confocal immunofluorescence of xenograft cryostat sections examined after 1 h of D-Prop injection showed redistribution of EGFR from the cell surface to perinuclear recycling endosomes, colocalizing there with TfnR and likely impeding its recycling back to the plasma membrane (Figure 4F,G). EGFR internalization/arrest at recycling endosomes, AKT signaling abrogation and mutp53 decreased levels, likely converge to reduce cell proliferation and increase apoptosis. These in vivo experiments also revealed an antiangiogenic effect of D-Prop reflected in lower CD31 staining. All these results suggest that D-Prop may be an effective treatment against tumors expressing EGFR and GOF mutp53.

## 4. Discussion

D-Prop emerges here as an anti-tumor drug able to counteract EGFR and mutp53-driven malignant traits [19,21]. We first show that tumor cell lines expressing “DNA-contact” (R273H) or “conformational” (R282W) p53 missense mutations internalize and accumulate EGFR in perinuclear recycling endosomes under D-Prop treatment. D-Prop also decreases AKT activity, viability and invasive migration in these cells. Surprisingly, D-Prop also reduces the levels of mutp53 protein by interfering with its HSP90 protective interaction, which is a recognized potential strategy against mutp53 oncogene addiction [3,10]. In vivo, D-Prop elicits similar effects, resulting in decreased tumor growth and prolonged mice survival.

EGFR endocytic trafficking towards recycling versus degradation routes is tightly intertwined with signaling intensity and therefore impact upon cell proliferation, survival and migration processes usually altered in cancer [7,8,9,23]. The EGFR predominantly follows recycling pathways when activated by low EGF concentrations [26] or high levels of TGF-α [19,25,55], or when Rab25, Rab11, RCP, Dyn-1 or GOF mutp53 become overexpressed by tumor cells [23,66]. GOF missense mutants of p53 and highly mitogenic TGF-α promote EGFR recycling through the Rab11 effector Rab-coupling protein (RCP) [19,25,55] and potentiate oncogenic signaling via ERK or AKT pathways [23,66,67,68,69]. Intense AKT signaling sustained by EGFR recycling enhances cell proliferation, survival and invasive migration [19,35,67], and also stimulate recycling of EGFR from peripheral APPL1 endosomes establishing a positive feedback [35]. The PA/PDE4/PKA pathway triggered by propranolol has been shown to induce clathrin-dependent and clathrin–independent EGFR endocytosis followed by EGFR accumulation at perinuclear endosomes [20,36]. The return of the EGFR to the cell surface after discontinuing the propranolol treatment indicates that the receptor accumulates at perinuclear endosomes due to recycling arrest, which can be reversible [36]. D-Prop also promotes diversion of EGFR activated by high ligand concentrations from the lysosomal-degradation pathway towards TfnR-containing recycling endosomes [36]. Here, we show that these effects are extensive to mutp53-expressing cells.

The EGFR colocalizes with TfnR in recycling endosomes during D-Prop treatment of GOF mutp53 cells, not only without adding exogenous ligand but also under high EGF or TGF-α stimulation. The functional consequences are those expected for a recycling arrest of EGFR in cells expressing GOF mutp53. We find that D-Prop remarkably abrogates AKT activation in response to both EGF and TGF-α in mutp53 cells. ERK activation also decreased but not as low as AKT and mostly in EIH1299-R273H and G-415-R282W, remaining unaffected in Panc-1-R273H cells. EGFR arrested at perinuclear endosomes likely disrupts the described intertwined AKT signaling and EGFR recycling [35]. We show that D-Prop counteracts the enhanced invasive migration of GOF mutp53 cells, previously associated with an elevated EGFR/α5β1 integrin recycling [19,55]. Integrins bind to the extracellular matrix while recycling EGFR focalizes signaling to cell regions engaged in directional motility [67,70,71].

Another property of GOF mutp53 is the resistance to gefitinib and erlotinib [63], shown here in cells that express R273H and R282W mutp53. D-Prop decreased the viability and increased apoptosis of cells expressing either R273H or R282W mutp53, despite their resistance to TKIs. Furthermore, EIH1299 induced to express mutp53 R273H acquired both invasive-migration and TKI-resistant properties that were counteracted by D-Prop. Considering the short half-life of about 4h reported for circulating propranolol in vivo [64,65], it is worth to remark that these effects occur with D-Prop treatment for just one hour twice a day, leaving periods of 6 and 16 h without the drug. Therefore, short periods of D-Prop treatment are enough to counteract malignancy traits of mutp53 cells such as invasive migration and TKI resistance.

Perturbing endocytic trafficking with D-Prop has been previously suggested as strategy to counteract the oncogenic influence of EGFR overexpression or activating mutations [20]. We have previously shown that most of the EGFR internalized in the absence of ligand return to the cell surface after washing out the drug [36]. However, whether the ligand-activated EGFR would return to the cell surface or become degraded if the drug is removed was not explored. This active receptor presumably signals from internal compartments while it remains accumulated in recycling endosomal compartments. Internalization of inactive EGFR and diversion of ligand-activated EGFR from the lysosomal-degradation pathway resulting in their arrest at recycling endosomes might be particularly deleterious to tumor cells that crucially depend on EGFR function [20,72]. For instance, HeLa cells and MKN45 gastric cancer cells transfected to overexpress EGFR display higher sensitivity to drugs that trigger the PA/PDE4/PKA pathway, compared with their non-transfected counterparts [20]. The NSCLC cell line H1975 expressing the double mutant EGFR^L858R/T790M^, which is oncogenic and resistant to TKIs, are also highly sensitive to this pathway, while non-tumor MDCK cells are unaffected [20]. The inaccessibility of EGFR to ligand and the accumulation of activated EGFR in recycling endosomes might counteract the “oncogenic addiction” to EGFR and GOF mutp53 activities [12,16].

Mutp53 proteins become hyper-stabilized, displaying high expression levels in cancer cells through protective interactions with heat shock protein chaperones, mainly HSP90, which avoid mutp53 ubiquitination by MDM2 and CHIP E3-ubiquitin ligases [10,34]. Disrupting the HSP90-mutp53 complex releases mutp53 and reactivates MDM2 and CHIP leading to mutp53 degradation [73]. The decrease of mutant p53 levels slow cell division and selectively decrease viability in mutp53 cancer cells [10,16,34], thus offering a therapeutic vulnerability. 

Strikingly, we unexpectedly found that D-Prop treatment for just 1 h decreases close to 50% the mutp53 levels assessed 24 h later, affecting both DNA-contact R273H and conformational R282W mutp53. This effect includes EIH1299 cells expressing R273H mutp53 under an inducible promoter and is abrogated by proteasome inhibition, indicating that D-Prop activates a mutp53 degradation pathway. Co-immunoprecipitation assays point to HSP90 as an effector of this pathway. HSP90 is a PKA substrate [42] and we find that D-Prop treatment decreases its phosphorylation by PKA and releases its interaction with mutp53. This suggests that HSP90 protective interaction with mutp53 is regulated by PKA activity, thus opening new possibilities of pharmacological reduction of mutp53 levels, including the use of D-Prop.

Xenografts of G-415-R282W GBC cells in NSG mice reproduced most D-Prop effects in vivo. Gallbladder cancer is a very aggressive neoplasia, displaying low responses to cytotoxic therapies such as Gemcitabine and Cisplatin [74]. Survival rate at 5 years is 5% [75]. Indeed, G-415-R282W cells are characterized by rapid growth and high malignancy [52]. Our results show that a single daily dose of D-Prop decreases tumor growth and extends mice survival. Immunohistochemistry analysis demonstrated decreased activity of AKT and ERK, lower levels of mutp53, internalization of EGFR, lower proliferation and increased apoptotic markers. The endothelial marker CD31 indicated a decreased angiogenesis, which is congruent with a described interference of D-Prop with angiogenic SOX18 transcriptional activity [76]. Median survival increased 67.8% (from 28 to 47 days; *n* = 5) and 42.5% (from 20 to 28.5 days; *n* = 10) under PO and IP D-Prop treatment, respectively (Figure 4B,D). As *TP53* mutations are common in GBC [77,78], these results might prompt the consideration of D-Prop as a plausible alternative in this particularly resistant cancer.

## 5. Conclusions

Propranolol in its L and D racemic formulation [40,79] has long been tested as beta-blocker in cancer to decrease stress-driven beta-adrenergic contribution to tumor growth and immunosuppression [80,81,82]. D-Prop has 60-100-fold less beta-blocker activity than L-propranolol [40,79], but is equivalent in triggering PA/PDE4/PKA signaling and EGFR internalization in cancer cell lines [20,37]. This allows the use of D-Prop at the higher doses, as required to trigger the PA/PDE4/PKA pathway involved in EGFR endocytosis. Remarkably, an old study of D-Prop as antiarrhythmic in human patients used four-fold [41] the single dose shown here to be effective against tumor growth. Drug-repurposing strategies ideally targeting two or more different oncogenic pathways [83], might be fulfilled by D-Prop in different cancers, either alone or in combination with other drugs. The dual effect of D-Prop internalizing EGFR and destabilizing mutp53 might generate a synthetic lethal-like condition, which could be quite promising as anti-tumor strategy.

## Data Availability

Not applicable.

## Supplementary Materials

The following are available online at https://www.mdpi.com/article/10.3390/cancers13143622/s1, Figure S1: EGFR colocalization with Rab11 under D-Prop treatment in G-415-R282W cancer cells, Figure S2: uncropped blots of Figure 2, Figure S3: Uncropped blots of Figure 3.

## Appendix A

*Antibodies and reagents.* D-Propranolol (Sigma-Aldrich, #P0689), EGF (R&D Systems, #236-EG), TGF-α (Thermo Fisher Scientific, #PHG0051), ponasterone A (Abcam, #ab144330), and Fibronectin (Sigma-Aldrich, #F2006) were used as indicated. Antibody source include: mouse monoclonal anti-EGFR (ATCC, #HB-8506); goat polyclonal anti-TfnR (CD71) (Santa Cruz Biotechnology, #sc-7088), mouse monoclonal anti-Lamp1 (Santa Cruz Biotechnology, sc-20011), rabbit polyclonal anti-phospho-EGFR (Tyr1068) (Cell Signaling Technology, #3777), rabbit polyclonal anti-EGFR (anti-EGFR984, [38]), rabbit monoclonal anti-phospho-ERK1/2 (Cell Signaling Technology, #4370), rabbit polyclonal anti-ERK1/2 (Cell Signaling Technology, #4695), rabbit monoclonal anti-phospho-AKT (Cell Signaling Technology, #4060), mouse monoclonal anti-AKT (BD Biosciences, #610861), rabbit polyclonal anti-p53 (Cell Signaling Technology, #9282), rabbit monoclonal anti-HSP90 (Cell Signaling Technology, #4877); rabbit monoclonal anti-Phospho-PKA Substrate (RRXS*/T*) (Cell Signaling Technology, #9624), mouse monoclonal anti-β-actin (Abcam, #ab8226), rabbit polyclonal anti-Ki-67 (Thermo Fisher Scientific, #PA5-19462), rabbit polyclonal anti-vimentin (Thermo Fisher Scientific, PA5-27231), mouse monoclonal anti-CD31 (Abcam, ab9498) and mouse monoclonal anti-cytokeratin (Abcam, ab7753); all antibodies were used according to manufacturer instructions.

*EGFR endocytosis analyzed by FACS.* Cells were incubated with D-Prop 100 μM for 30 min at 37 °C, washed twice with ice-cold DPBS at 4 °C and incubated with monoclonal antibody against the extracellular domain of EGFR 1:20 (ATCC, #HB-8506) in “binding buffer” (MEM Hank`s balanced salts, HEPES 1 mM and BSA 0.2%) for 45 min at 4 °C with gently shaking. Cells were washed twice with ice-cold DPBS and then incubated with anti-mouse Alexa-488 secondary antibody (1:500) for 1-h at 4 °C with gently shaking. After washing with DPBS, cells were incubated with pre-heated EDTA 5mM at 37 °C for 10 min, centrifuged at 1100g for 3 min and washed three times with FACS buffer (PBS with 2% FBS). The cells were then fixed in Cytofix (BD Biosciences, #554655) and subjected to flow cytometry analysis using a FACSCalibur flow cytometer with CellQuest software (BD Biosciences). Relative percentage of EGFR at cell surface was calculated as follows = (Mean Fluorescence—Mean Fluorescence of cells incubated only with secondary antibody)/(Mean Fluorescence of control (time 0)—Mean Fluorescence of cells incubated only with secondary antibody) × 100.

*MTT viability assay.* Cells treated as indicated were incubated with MTT (5 mg/mL) for 2 h at 37 °C. MTT was solubilized in 0.02 N HCl 4 mM and 0.1% NP-40 in isopropanol and absorbance was measured at 590 nm with a Synergy LX Multi-Mode Reader (BioTek Instruments).

*Inverted Invasion Assay.* As previously described [46,47], Matrigel (Corning, #35423) 5 mg/mL, mixed with fibronectin (25 μg/mL), was polymerized in 24-well Transwell™ polycarbonate filters 8-μm pore size inserts (Corning, #3422) for 1 h at 37 °C. Inserts were inverted, and 8 × 104 cells were seeded to the bottom of the filter and allowed to adhere for 4–5 h at 37 °C in 5% CO_2_ before returning to the right side up. Serum free media was added to the wells of the transwell plate and media with FBS 10% plus 20 ng/mL EGF was added in the upside of the chamber on top of the Matrigel plug. After 5 days of daily incubation with D-Prop (100 μM) for 1 h twice a day, Matrigel plugs were fixed with 4% PFA, treated with 0.2% Triton X-100. and stained with DAPI. Cells at the bottom of the filter that failed to migrate through the filter were removed using tissue. Images at 10-μm intervals were taken with a spectral confocal microscope (SP8, Leica) using the 10X objective. ImageJ software (NIH) was used to determine the integrated density of each image section to calculate the invasion index = (∑ integrated density of first 30 µM)/(∑ integrated density of invasion) expressed as a fold change.

*Immunoprecipitation and immunoblotting.* Cells were lysed in ice cold immunoprecipitation buffer (25 mM HEPES, 150 mM NaCl, 1 mM MgCl2, 0.4% NP-40), supplemented with an antiprotease mixture (4mM PMSF, 4 μg/mL pepstatin, 4 μg/mL leupeptin and 1 mM sodium orthovanadate) at 4 °C. Protein lysates were clarified by centrifugation. Immunoprecipitations experiments were performed using equivalent protein amounts at 4 °C for 1h using the respective primary antibodies previously bound to 30 μL of Protein A-Sepharose beads (SCBT). Beads were washed with ice cold immunoprecipitation buffer (25 mM HEPES, 150 mM NaCl, 1 mM MgCl2, 0.4% NP-40) and then incubated at 95 °C for 5 min in standard Laemmli loading buffer. Proteins lysates for immunoprecipitation and western blotting were separated by SDS-PAGE on 7.5 or 10% acrylamide gels, transferred to PVDF membranes (Thermo Fischer Scientific, # PI88520) and then subjected to standard immunoblotting procedures previously described [48].

*Subcutaneous xenografts.* G-415 cancer cells (2 × 10^6^) were transplanted subcutaneously with 50% Matrigel to NOD/SCID/IL2rγ^null^ (NSG) immunodeficient mice (Jackson Laboratory), as described [52]. Tumor growth was monitored daily using digital caliper measurements. When tumor growth reaches 50 mm^3^, NSG mice were subjected to either oral gavage (PO) (*n* = 5) or intraperitoneal (IP) (*n* = 10) treatment with D-Prop 70 mg/kg/day and 20 mg/kg/day for indicated times, respectively. PBS was used as a control vehicle. Survival analysis was performed by the Kaplan–Meier method and log-rank analysis.

*EGFR internalization in G415 xenografts.* NSG mice transplanted with G-415 cancer cells (2 × 106) were treated with intraperitoneal (IP) injections with D-Prop 20 mg/kg/day or control vehicle (PBS) once tumor growth reaches 50 mm^3^. After 5 days of treatment the mice were fasted for 3 h, treated IP with D-Prop for 1 h, euthanized and perfused with PAF 4%. Subcutaneous xenografts were removed and cryoprotected for 24 h in 30% sucrose. Tissue was then embedded in OCT and 5-μm slices were obtained using a Cryostat (Leica, Germany). Tissue sections were subjected to indirect immunofluorescence for EGFR and TfnR. Hoechst was used to stain nuclei. Images were obtained using a SP8-Leica spectral confocal microscope.

*Immunohistochemical analysis.* Formalin-fixed, paraffin-embedded sections of G-415 xenografts from D-Prop-treated NSG mice were processed for immunohistochemistry as previously described [84]. Sections were incubated with indicated primary-antibodies all individually diluted in 10 mM Tris-HCl buffer (pH 7.8) and 1 % BSA (*w*/*v*). Then sections were incubated with secondary HRP-conjugated either anti-rabbit IgG or anti-mouse IgG (Rockland Immunochemicals) for 2 h at room temperature. Hematoxylin/Eosin was used as a nuclear and cytoplasm counterstain in tissue sections, respectively. IHC image analyses for quantification of cellular positivity of the markers above detailed, was made manually using 3–6 images per sample depending on tumor size and excluding necrotic areas.
