# Supplementary figures and images for: D-Propranolol Impairs EGFR Trafficking and Destabilizes Mutant p53 Counteracting AKT Signaling and Tumor Malignancy
Source: Cancers (Basel). 2021 Jul 20;13(14):3622. doi: 10.3390/cancers13143622 (PMC8305715; doi:10.3390/cancers13143622)

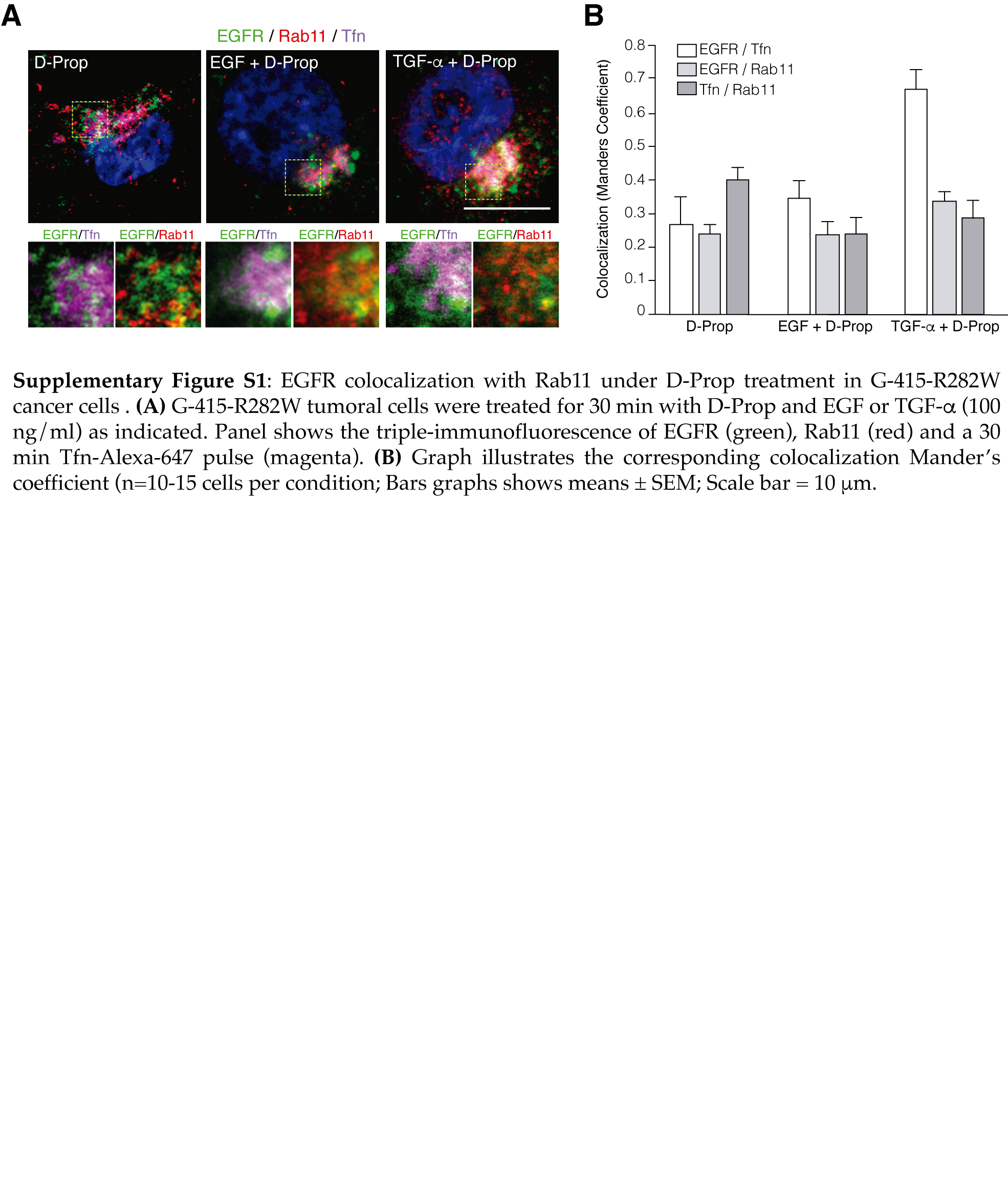

Supplement: Supplementary file 1 [file cancers-13-03622-s001.zip › Figure S1.tif]

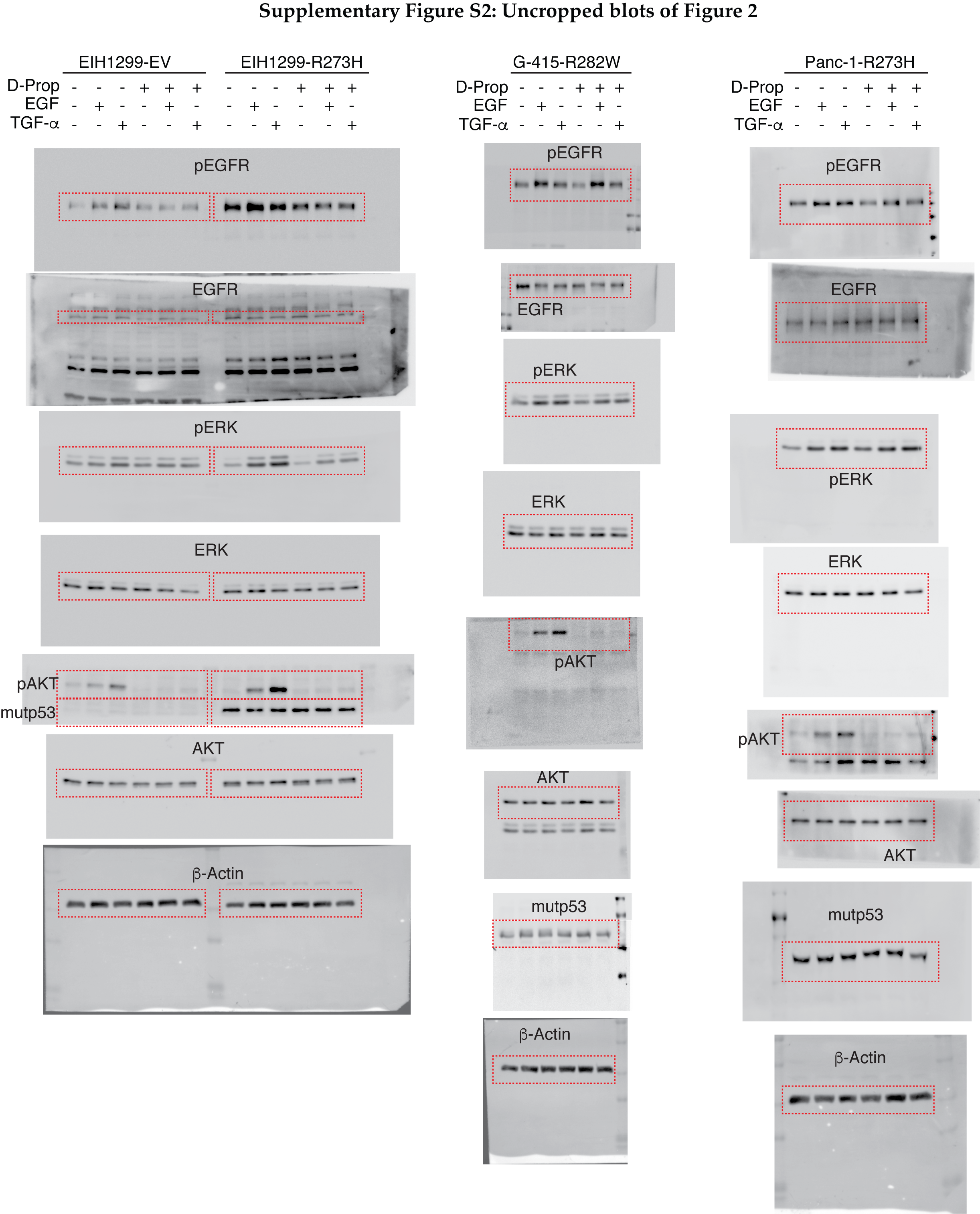

Supplement: Supplementary file 1 [file cancers-13-03622-s001.zip › Figure S2.tif]

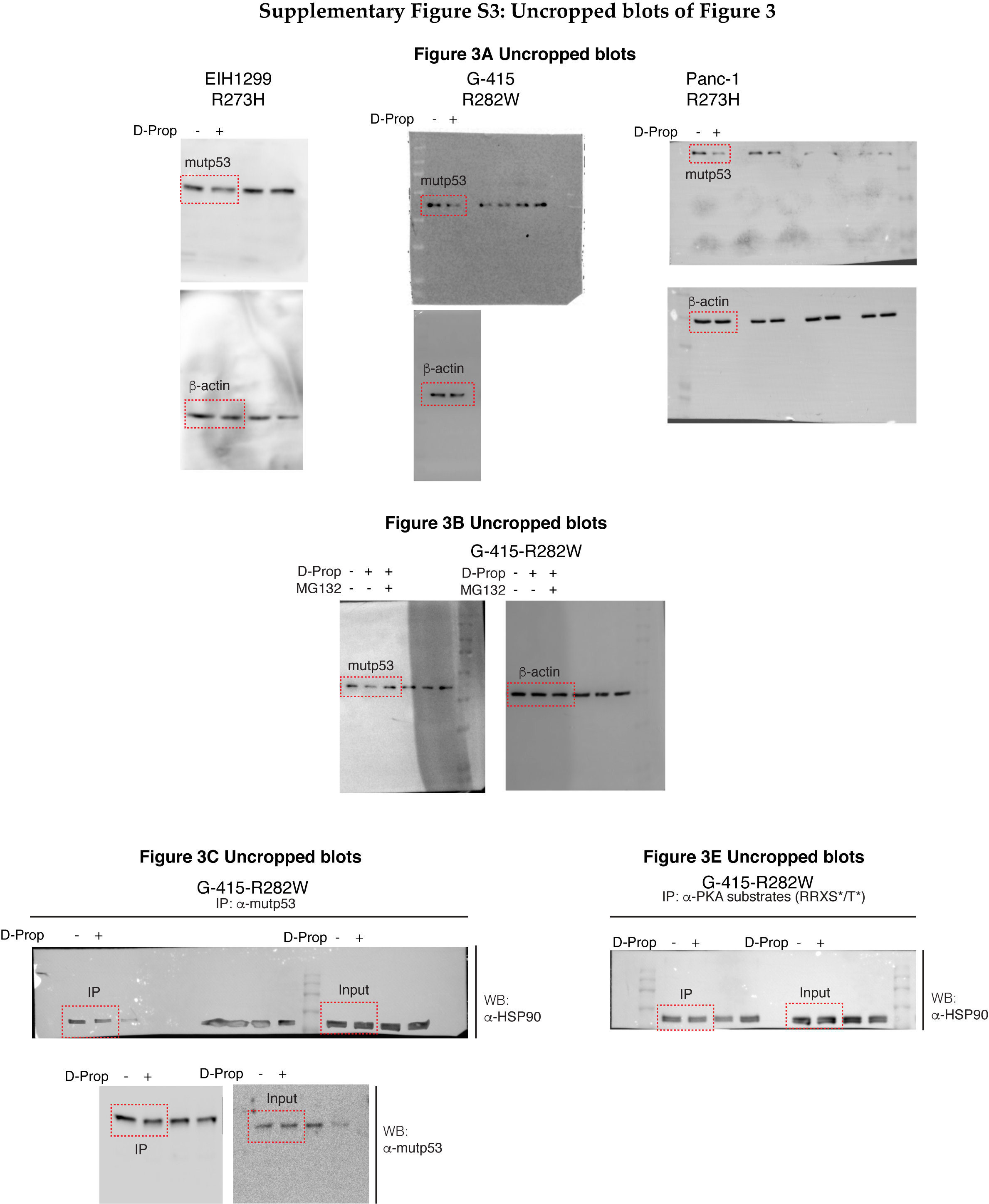

Supplement: Supplementary file 1 [file cancers-13-03622-s001.zip › Figure S3.tif]
